# Physiological Predictors of Competition Performance in CrossFit Athletes

**DOI:** 10.3390/ijerph17103699

**Published:** 2020-05-24

**Authors:** Rafael Martínez-Gómez, Pedro L. Valenzuela, Lidia B. Alejo, Jaime Gil-Cabrera, Almudena Montalvo-Pérez, Eduardo Talavera, Alejandro Lucia, Susana Moral-González, David Barranco-Gil

**Affiliations:** 1 Faculty of Sport Sciences, Universidad Europea de Madrid, 28670 Madrid, Spain; martinezgomezrafael2015@gmail.com (R.M.-G.); jaime.gil@universidadeuropea.es (J.G.-C.); almudena.montalvo@universidadeuropea.es (A.M.-P.); edutalfer@gmail.com (E.T.); alejandro.lucia@universidadeuropea.es (A.L.); susana.moral@universidadeuropea.es (S.M.-G.); david.barranco@universidadeuropea.es (D.B.-G.); 2 Department of Systems Biology, University of Alcalá, 28805 Madrid, Spain; pedrol.valenzuela@edu.uah.es; 3 Department of Sport and Health, Spanish Agency for Health Protection in Sport (AEPSAD), 28016 Madrid, Spain; 4 Instituto de Investigación Hospital 12 de Octubre (imas12), 28009 Madrid, Spain

**Keywords:** sport, VO_2max_, strength, power, laboratory tests, jump

## Abstract

The aim of this study was to determine the physiological variables that predict competition performance during a CrossFit competition. Fifteen male amateur CrossFit athletes (age, 35 ± 9 years; CrossFit experience, 40 ± 27 months) performed a series of laboratory-based tests (incremental load test for deep full squat and bench press; squat, countermovement and drop jump tests; and incremental running and Wingate tests) that were studied as potential predictors of CrossFit performance. Thereafter, they performed the five Workouts of the Day (WODs) corresponding to the CrossFit Games Open 2019, and we assessed the relationship between the laboratory-based markers and CrossFit performance with regression analyses. Overall CrossFit performance (i.e., final ranking considering the sum of all WODs, as assessed by number of repetitions, time spent in exercises or weight lifted) was significantly related to jump ability, mean and peak power output during the Wingate test, relative maximum strength for the deep full squat and the bench press, and maximum oxygen uptake (VO_2max_) and speed during the incremental test (all *p* < 0.05, r = 0.58–0.75). However, the relationship between CrossFit Performance and most laboratory markers varied depending on the analyzed WOD. Multiple linear regression analysis indicated that measures of lower-body muscle power (particularly jump ability) and VO_2max_ explained together most of the variance (R^2^ = 81%, *p* < 0.001) in overall CrossFit performance. CrossFit performance is therefore associated with different power-, strength-, and aerobic-related markers.

## 1. Introduction

CrossFit is a strength and conditioning exercise program aiming at increasing work capacity across several physical domains (endurance, strength, flexibility) by using ‘functional’ movements. It thus combines different ‘tasks’ such as weightlifting, gymnastics, and traditional ‘aerobic’ exercise modalities (e.g., running, rowing, cycling). These tasks are combined in a specific manner for each of the different types of workout sessions, which are known as “Workout of the Day” (WOD) [1]. On the other hand, performance in a given task is usually assessed by measuring the time needed to finish the task in question, or by determining the number of repetitions performed or the total weight lifted. However, despite the increasing popularity of CrossFit [1] and CrossFit competitions (known as ‘Opens’ and consisting of five WODs that athletes were previously unfamiliar with and are performed consecutively during one month) little is known about the physiological determinants of performance in this sport [2,3,4,5].

Laboratory-based measures related to endurance (e.g., as assessed during an incremental running test until volitional exhaustion) or muscle strength/power (jump ability, such as countermovement (CMJ) or squat jump (SJ), and incremental loading tests for the assessment of maximum power and 1-repetition maximum (1RM)) have been reported as valid markers for predicting or monitoring performance in a variety of sports [6,7,8,9,10,11]. There is, however, little information on how these measures relate to CrossFit performance, which would provide insight into the main physiological determinants of this sport, thereby allowing coaches and athletes to optimize training programs. Some authors have found a relationship between markers of maximal aerobic capacity (e.g., maximum oxygen uptake (VO_2max_)) and CrossFit performance [2,4,5]. Others have reported that the strongest (e.g., with the highest 1RM) and most powerful athletes (e.g., with the highest power values during the deep full squat exercise or the Wingate Anaerobic Test (WAnT)) achieve the highest CrossFit performance [2,3,5]. Additionally, one study showed that faster recovery ability between continuous WAnT trials correlated to highest CrossFit performance [12]. However, the level of association seems to depend on the type of WOD analyzed [2,4,5]. Thus, some WODs seem to be associated with ‘aerobic’-related measures while others are more associated with power/strength-related measures. However, further evidence is needed in this regard. In addition, to the best of our knowledge no previous study has assessed the physiological determinants of CrossFit performance during actual competition (as opposed to ‘standardized’ WODs athletes were previously familiarized with).

The scarcity of evidence on which tests or physiological variables correlate with CrossFit performance might be due, at least partly, to the wide variety of ‘domains’ included in CrossFit WODs (e.g., strength, power and aerobic-related exercises) coupled with the paucity of studies on this topic. In this context, the aim of the present study was to determine which physiological variables could predict performance during a CrossFit competition (The Open, 2019), by analyzing markers of ‘aerobic’ and ‘anaerobic’ capacity, strength, and power. Given the variety of WODs included in CrossFit Opens and the complexity of the different exercises we hypothesized that CrossFit performance would be associated with a combination of different fitness capacities (ranging from ‘aerobic’ to strength/power measures).

## 2. Materials and Methods

### 2.1. Experimental Design

**Figure 1:**
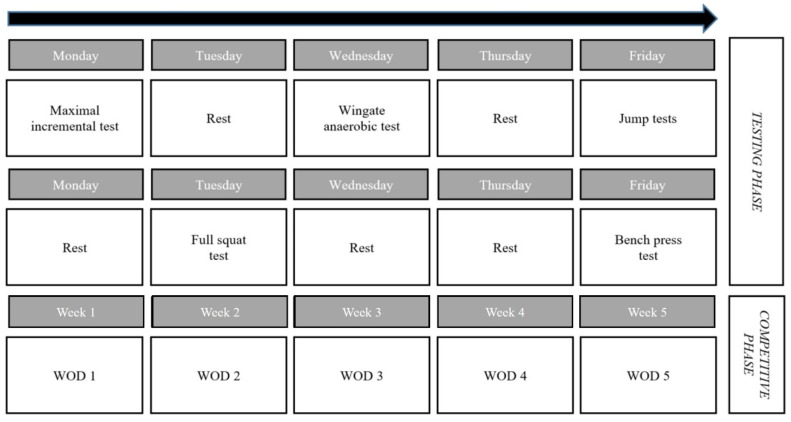
Schematic representation of the study protocol. Abbreviation: WOD, workout of the day.

The present study followed a cross-sectional design. During the two weeks prior to the start of the competitive phase (i.e., the CrossFit Games Open, 2019) and after a familiarization session, participants performed—consistently in the same order—a series of laboratory tests aimed at assessing potential markers of endurance (incremental maximal running test) and power/strength-related performance (incremental load test for deep full squat and bench press, jump tests, and WAnT). This allowed us to analyze/validate their potential as predictors of CrossFit performance. One week after the last test, all participants performed the five WODs of the CrossFit Games Open 2019 on five different days in a CrossFit ‘box’ (i.e., a gym) for one month, on the same day and consistently in the same order (Figure 1). Athletes had one attempt to complete each WOD. Athletes were familiarized with each of the proposed exercises but not with how they were specifically combined for each of the WODs. Participants were required to rest for a minimum of 24 h before each test/WOD to avoid fatigue.

### 2.2. Subjects

278 recreationally trained men from a local CrossFit center were eligible to participate in our study. Inclusion criteria were ≥1-year experience in CrossFit, training ≥ 3 times per week during the preceding year, being familiar with each of the exercises included during the WODs, and being able to attend all testing sessions and WODs during the study. In total, 31 men met the inclusion criteria, of which 15 volunteered to participate. During the study, participants maintained their regular training program and dietary pattern, but were required to refrain from exercising at least one week before each testing session or WOD, and from consuming ergogenic aids or stimulants (e.g., creatine, caffeine) during this period. The study was approved by the Institutional Review Board of “Hospital Universitario Fundación Alcorcón” (19/51). Participants were informed of the benefits and risks of the investigation and provided written informed consent. All procedures were conducted following the standards set by the Declaration of Helsinki and its later amendments.

### 2.3. Measures

#### 2.3.1. Lower- and Upper-Body Strength and Power Tests

Participants performed an incremental load-free test (i.e., not performed on a guided machine) for both the deep full squat and bench press exercises. Bar mean propulsive velocity (MPV) during the concentric phase was measured with a linear position transducer (Chronojump, Boscosystem, Spain), and power was calculated based on the total mass moved (i.e., sum of the subject’s body mass and the external load for the deep full squat, vs. only the external load for the bench press). The linear position transducer has been previously validated for the measurement of bar velocity [13]. The initial weight was 20 kg (i.e., only the bar), and the load was increased by 15 kg until a constant decrease in MPV was observed (i.e, from 0.69 to 0.60 m∙s^−1^) and subjects were closer to the minimum MPV; thereafter, the load was increased by 10 kg. Tests were deemed concluded when MPV decreased to 0.6 m∙s^−1^ for the deep full squat [13] and 0.4 m∙s^−1^ for the bench press [14]. A three-minute rest was allowed between loads. Athletes performed three repetitions with each load, and the best result (based on the mean concentric propulsive power) was used for analysis. The maximum mean concentric propulsive power (Pmax) registered during the incremental test was used for analysis as absolute (W) and relative (W∙kg^−1^) values.

To avoid excessive physical stress, the 1RM was estimated for each exercise based on the individual force-velocity relationship through linear interpolation, assuming that it was attained with an MPV of 0.30 m·s^−1^ for the deep full squat [14] and 0.16 m·s^−1^ for the bench press [15]. According to recent studies, this method provides an accurate estimate of the actual 1RM [16,17]. We checked that the linear regression accurately fitted the load-velocity data by examining the correlation coefficients (R^2^ = 0.97 ± 0.02 and 0.98 ± 0.02 for the deep full squat and the bench press, respectively). The 1RM was expressed both as absolute (kg) and relative (% of body weight) values.

#### 2.3.2. Jump Performance

Jump performance was measured using an optoelectric cell system (Optojump, Microgate, Bolzano, Italy) while participants performed SJ, CMJ, and drop jumps (DJ). The instrument used for the measurement of jump height has proven valid and reliable compared with force plates [18]. Participants performed three trials for each type of jump, and the mean of the three trials was used for analysis. Participants were instructed to place their hands on their hips while performing the jumps. During the SJ, they performed a downward movement to reach 90° of knee flexion, stopped at that position for ~2 s, and then tried to achieve the maximum jump height without performing any countermovement. The same procedure was performed during the CMJ, but no stop was made at 90° of knee flexion and countermovement was allowed. For the DJ, participants stepped from a 40-cm-high bench and jumped as high as possible with the minimal possible ground contact time. Reactive strength index (RSI) was calculated as jump height in the DJ divided by contact time. During all jumps, participants were instructed not to flex their knees during flight or landing phases to avoid overestimation of flight time.

#### 2.3.3. Maximal Incremental Test

Participants performed a maximal incremental running test on a treadmill (HP Cosmos Quasar, Nussdorf-Traunstein, Germany) for the determination of the first (VT1) and second (VT2) ventilatory thresholds, and VO_2max_. After a 3-min warm-up at 5 km·h^−1^, the test started at 6 km·h^−1^ and speed was increased by 0.25 km·h^−1^ every 15 s until volitional exhaustion, keeping the inclination steady at 1% during the entire test [19]. Gas exchange data were collected continuously using a breath-by-breath system (Ultima Series Medgraphics, Cardiorespiratory Diagnostics, Saint Paul, MN, USA). VT1, VT2 and VO_2max_ were determined as described [20]. Peak velocity (V_peak_) was defined as the highest velocity attained during the test. We also assessed the mean muscle oxygen saturation (SmO_2_) of the right *vastus lateralis* during the incremental test by means of near infrared spectrometry (Humon, Cambridge, MA, USA) [21].

#### 2.3.4. Wingate Anaerobic Test

Participants performed the WAnT on a cycle-ergometer (Monark, 818 E, Varberg, Sweden) as explained elsewhere [21]. After a standardized warm-up (pedaling with a resistance of ~2% of their body weight and a cadence of 70–90 rpm for 5 min), they completed a 30-s all-out test with a resistance of 7.5% of their body weight [21]. The mean (MPO) and peak (PPO) power output were determined as the average PO attained during the test and the highest PO achieved during 3 consecutive seconds, respectively. The fatigue slope (FS) was computed with the following equation [21]:

FS(%) = ((PPO − Final PO) / (PPO) × 100

The mean SmO_2_ of the right *vastus lateralis* muscle was measured during the WAnT as described for the incremental running test. Fingertip capillary blood samples (0.5 μL) were taken at baseline, and at 0, 3, 5 and 10 min after the test, and lactate concentration was quantified using a portable lactate analyzer (Lactate Scout, SensLab GmbH; Leipzig, Germany). The highest lactate value recorded for each participant was considered as the lactate peak ((La^−^)_peak_) [22,23].

#### 2.3.5. CrossFit Performance

The specific details of the five WODs used in this study, known as 19.1, 19.2, 19.3, 19.4 and 19.5, can be seen at [24]. They are briefly explained below:

- WOD 1. Participants had 15 min to complete as many rounds as possible of 19 wall-ball shots (20-lb ball to a 10-foot target) and 19 calories of rowing.
- WOD 2. Participants had 8 min to complete 25 toes-to-bar, 50 double-unders, 15 squat cleans (135 lb), 25 toes-to-bar, 50 double-unders, and 13 squat cleans (185 lb). If they completed these exercises before the 8-min mark, 4 further minutes were added and they had to perform 25 toes-to-bar, 50 double-unders, and 11 squat cleans (225 lb). If they completed again these exercises under 12 min, 4 min were added and they had to perform 25 toes-to-bar, 50 double-unders, and 9 squat cleans (275 lb). If completed under 16 min, 4 additional minutes were added and they performed 25 toes-to-bar, 50 double-unders, and 7 squat cleans (315 lb). The maximum time allowed was 20 min.
- WOD 3. Participants had a maximum of 10 min to complete 200-foot dumbbell (50 lb) overhead lunges, 50 dumbbell (50 lb) box step-ups (24-inch box), 50 strict handstand push-ups, and 200-foot handstand walk in the minimum time possible.
- WOD 4. Participants had a maximum of 12 min to complete 3-rounds of 10 snatches (95 lb) and 12 bar-facing burpees in the minimum time possible. They then rested for 3 min and continued with 3 rounds of 10 bar muscle-ups and 12 bar-facing burpees, which they had to complete in the minimum time possible.
- WOD 5. Participants had 20 min to perform 33 thrusters (95 lb) and 33 chest-to-bar pull-ups, followed by 27, 21, 15, and 9 reps of the same sequence (i.e., same number for thrusters and chest-to-bar pull-ups).

The number of repetitions performed on each WOD was used for analysis. Participants were then ranked in positions from 1 to 15 depending on their performance (number of repetitions) in each WOD. They also received a score after each WOD based on their classification within the group (one point for the first position, two points for the second one, and so on), and were ranked for overall CrossFit performance considering the sum of the scores attained in the five WODs (with lower scores reflecting a better performance). Only those participants who performed all the WODs were included in analyses. Participants were divided by the median into a low (LP) and a high-performance (HP) group attending to the final ranking as described elsewhere [3,25,26].

#### 2.3.6. Statistical Analysis

In a previous study [4],we observed significant and large differences (effect size [ES] of 1.58–1.66) for 1RM between those individuals who attained the best and worst performance during different CrossFit WODs. Based on these results, we used GPower (version 3.1.9.2, Universität Düsseldorf, Germany) to estimate that a sample size of 12 participants would be sufficient to find significant differences between groups (ES = 1.55, power > 80%, one tail α < 0.05).

Normal distribution (Shapiro–Wilk test) and homoscedasticity (Levene’s test) of the data were confirmed before any statistical treatment. Simple linear regression analyses were performed to assess the relationship between each variable and CrossFit performance on each WOD (expressed as number of repetitions performed) and overall CrossFit performance (i.e., sum of the scores attained in the five WODs), computing Pearson’s correlation coefficients (r). Spearman’s rank correlation coefficients (ρ) were calculated to analyze the relationship between each variable and the position (ranking (first, second, and so on)) within the group. Correlation coefficients of 0.1, 0.3, 0.5, 0.7 and 0.9 were considered small, moderate, large, very large and extremely large, respectively [27]. Least-squares multiple regression analysis was used to analyze the variables that appeared significantly correlated (uncorrected *p*-value < 0.05) with overall CrossFit performance, progressively removing those variables in the model that showed no significant association (i.e., going from the largest to the lowest *p*-value). Variance inflation factors (VIF) among the variables eventually included in the model were examined to inspect for multicollinearity and were set at a maximum of 5. The magnitude of the differences between groups was assessed through the computation of ES (Cohen’s d), which was considered as trivial (d < 0.2), small (d = 0.2–0.6), moderate (d = 0.6–1.2), large (d = 1.2–2.0) or very large (d = 2.0–4.0) [27]. Analyses were conducted with a statistical software package (SPSS 23.0, IBM; Armonk, NY) and an Excel Spreadsheet [28,29], setting an α-level of *p* < 0.05.

## 3. Results

Participants’ characteristics and differences between the HP and LP groups are shown in Table 1 and Table 2. No significant differences were observed for age, anthropometrical variables or training experience (*p* > 0.05) (Table 1).

Regarding jump performance, we only found statistical significance in SJ for the comparison between HP group and LP group (Table 2). The HP group also showed a higher PPO and MPO during the WAnT, but only when expressed in relative values (W/kg, Table 2), as well as a higher VO_2max_ and Vpeak (Table 2). No differences (*p* > 0.05) were observed, however, for vVT1 or vVT2. Lastly, the HP group showed a higher relative 1RM in the bench press exercise, but no between-group differences were found for absolute 1RM (in kg) or for Pmax in the deep full squat (Table 2).

The relationships between the variables assessed during laboratory tests (jump ability, lower- and upper-body strength/power, and ‘aerobic’ and ‘anaerobic’ markers) and the repetitions performed in each WOD are shown in Table 3. Markers of jumping ability were largely to very largely correlated with the number of repetitions performed in most WODs (Table 3). Relative mean PO during the WAnT was also significantly correlated with the number of repetitions in four of the five WODs, but no consistent associations were found for the remaining variables assessed on the WAnT. VO_2max_ and Vpeak were associated with the repetitions performed in two and four of the five WODs (Table 3). Finally, measures of relative strength in the squat and bench press exercises, and relative Pmax in the bench press exercise, were largely associated with the number of repetitions performed in three or more of the five WODs (Table 3).

Associations between the laboratory variables and the position within the group for each WOD are shown in Table 4. Jumping ability was the best predictor of CrossFit performance, with all jump-related variables (SJ, CMJ and RSI) largely associated with at least four of the five WODs performed, as well as with the final ranking (Table 4). The same trend was observed for the relative (W/kg)—but not absolute (W)—PPO and MPO during the WAnT, which were also largely related to performance in at least four WODs as well as to the final ranking (Table 4). VO_2max_ and Vpeak were also significantly and strongly related to performance in three WODs and to the final ranking (Table 4). Lastly, relative—but not absolute—upper- and lower-body maximal strength (i.e., 1RM for the bench press and the deep full squat, respectively, both corrected for body weight) were largely related to performance in at least three WODs as well as to the final ranking (Table 4). A significant relationship was also observed for the maximum power in the bench press exercise, but this relationship remained significant in only one of the five WODs.

Multiple regression analysis with those variables that appeared individually correlated showed that the combination of VO_2max_, SJ, and RSI was the simplest model that best explained CrossFit performance (i.e., final score summing all WODs), accounting for 81% of the performance variance (R^2^ = 80.7 %, *p* < 0.001) and with all variables significantly contributing to the model (VO_2max_, standardized *β* = −0.359; *p* = 0.026; SJ, *β* = −0.417, *p* = 0.029; RSI, *β* = −0.385, *p* = 0.043). There was no autocorrelation in the residuals (Durbin Watson statistic = 2.40, *p* = 0.786), which were normally distributed, and no signs of multicollinearity (VIF = 1.1–1.6). The following equation determined the relationship:

CrossFit performance (overall score) = 192.68 − 38.33 ∗ RSI (cm/ms) − 1.52 ∗ SJ (cm) − 1.31 × VO_2max_ (ml∙kg^−1^∙min^−1^)

The removal of any of these variables from the model resulted in an R^2^ value of 69%–72% (*p* < 0.001). However, the addition of other variables did not meaningfully improve the accuracy or significance of the model.

## 4. Discussion

The aim of the present study was to explore the relationship between CrossFit performance and several physiological markers related to ‘aerobic’ and ‘anaerobic’ capacity, strength, and power. Our results show that CrossFit performance is associated with a spectrum of physiological ‘domains’, including markers of power (jump ability and power during the WAnT), strength (1RM relative to body weight) and aerobic performance (VO_2max_ and Vpeak). Indeed, these variables—particularly jump performance and relative power during the WAnT—appeared as strong predictors of CrossFit performance in the vast majority of the WODs performed and the combination of power (SJ and RSI) and aerobic (VO_2max_) markers together explained most of the variance (81%) in overall CrossFit performance.

Research into CrossFit performance has grown exponentially in recent years, but there remains scarce information on the performance determinants of this sport [1]. In the present study, lower-body muscle power—as measured by jump ability and PPO during the WAnT—appeared as one of the strongest predictors of performance. Other studies have also found a relationship between lower-body muscle power and CrossFit performance. For instance, we recently observed that power-related indices measured in the squat test were related to a greater performance in most of the WODs analyzed [3]. Similarly, and in agreement with our findings, other authors recently reported that the PPO measured during a WAnT was related to performance in two of the four WODs analyzed [5]. Of note, some CrossFit exercises included in this study such as singles or double-unders involve repeated jumps, which might require high levels of lower-body muscle power. No relationship was found between SJ or CMJ and performance in WOD 1, which might be due to the nature of the exercises performed (row and wall ball). In turn, SJ, CMJ and RSI were strongly correlated to performance in WODs 2 and 5, which include exercises such as double-unders and thrusters, respectively. Moreover, jump ability has been related to performance in other exercises such as the loaded squat jump [26,30]. PPO measured during the WAnT is also related to key athletic actions including jumping or sprinting [30,31], which are usually present in CrossFit WODs. Thus, the assessment of lower-body muscle power can provide valuable information on CrossFit performance. It must be noted that, contrary to a recent study [3], we did not observe a clear relationship between power indices measured during the deep full squat and CrossFit performance, which might be due to the lower velocity attained during this exercise compared with other power actions such as the WAnT or unloaded jumps. Future research should confirm the validity of power measures obtained during the deep full squat for the prediction of CrossFit performance.

The relative—but not absolute—maximum strength of both the upper and lower limbs was related to CrossFit performance, which reflects the importance of body weight in most exercises (e.g., wall-ball shots, squat cleans, overhead lunges, handstand walk). In most CrossFit exercises, athletes not only have to lift or throw an external load, but also their own body mass. For this reason—as in other sports—trying to reach a balance between maximum strength and body mass will be of paramount importance [32], although for CrossFit the importance can likely differ depending on the WOD performed [2,3,5].

Lastly, an interesting finding of the present study was that both aerobic- and anaerobic-related markers were linked to CrossFit performance, as measured by the VO_2max_ and Vpeak during the incremental test and by the MPO or PPO during the WAnT. These results are in agreement with those of previous studies. For instance, Dexheimer et al. reported that VO_2max_ was related to performance in CrossFit WODs such as Fran, Nancy or Grace [5]. Other authors have also found a relationship between performance on the WAnT and performance in different types of WODs [2]. Different correlations were observed between PPO and VO_2max_ in WOD´s position and overall ranking, which is in apparent disagreement with a study showing that both aerobic capacity and anaerobic power were similarly associated with CrossFit performance [4]. This could be explained by the type of exercises that our participants had to perform in the different WODs, especially in WODs 2 and 3. Indeed, exercises in WOD 2 included a weighted movement where PPO has a domain instead of VO_2max_, and the same would apply to WOD 3—where athletes had to do 200-foot dumbbell (50 lb) overhead lunges, 50 dumbbell (50 lb) box step-ups (24-inch box), 50 strict handstand push-ups, and 200-foot handstand walk in the minimum time possible. It is worth noting that the importance of aerobic-related markers on CrossFit performance is often overlooked by athletes and coaches, who tend to focus on power and more anaerobic-like exercises, Thus, our findings support the inclusion of exercises aiming at improving both ‘aerobic’ and ‘anaerobic’ fitness.

In summary, the present findings show that CrossFit performance is associated with a variety of fitness markers related to both aerobic and anaerobic/power capabilities, information that might be useful to coaches in order to optimize training prescription. Our results thus underscore the complexity of this sport and support the importance of aerobic and strength training to potentially enhance CrossFit performance. Particularly, our results show that the combination of lower-body muscle power (as reflected by SJ performance), reactive strength (as reflected by the RSI during a DJ) and aerobic capacity (as measured with the VO_2max_) explains most of the variance of CrossFit performance, which might support focusing on improving these variables, for example, by performing loaded and unloaded plyometrics and other explosive movements, and high-intensity aerobic exercise, for the enhancement of CrossFit performance, or using these tests to assess CrossFit athletes during the season.

Some limitations of this study must be acknowledged, such as the low sample size, the use of multiple tests and a non-corrected *p*-value (which might increase the likelihood of type I error), and its cross-sectional nature, which precludes us from knowing whether enhancing any of the analyzed variables would result in an improved CrossFit performance. Moreover, whether these variables would be associated with CrossFit performance in non-experienced athletes remains unknown. In addition, as the characteristics and exercises of the CrossFit Open Games change each year, more studies including different WODs and larger sample sizes are needed to confirm our findings. It must be noted, however, that some of the analyzed markers were related to performance in WODs that included a great variety of exercises, which suggest that this association might also be observed in other WODs. Finally, the reliability of performance during WODs remains unknown and should be elucidated in future studies.

## 5. Conclusions

The present study shows that CrossFit performance is at least partly associated with power measures including jump ability and mean and peak PO during the WAnT, measures of relative strength including for both the upper- and the lower-limb, and markers of endurance performance including VO_2max_ and Vpeak. The combination of VO_2max_, SJ, and RSI explained most of the variance (~81%) in CrossFit performance, which might potentially support focusing on improving these variables for the enhancement of CrossFit performance, or using these tests as predictors of CrossFit performance.

## Figures and Tables

**Table 1:** Descriptive characteristics of study participants.

	All Subjects (*n* = 15)	HP (*n* = 7)	LP (*n* = 8)	*p*-Value	ES
Age (years)	35 ± 9	32 ± 10	38 ± 8	0.254	0.67
Height (cm)	177 ± 5	176 ± 4	178 ± 6	0.468	0.39
Weight (kg)	81 ± 9	78 ± 2	84 ± 11	0.161	0.73
BMI (kg∙m^2^)	25.9 ± 1.5	25.2 ± 0.6	26.5 ± 1.8	0.091	0.94

Data are Mean ± SD. Abbreviations: BMI, body mass index; ES, effect size; HP, high-performance group; LP, low-performance group.

**Table 2:** Differences between high (HP) and low-performance (LP) groups.

Test	Variable	HP (*n* = 7)	LP (*n* = 8)	*p*-Value	ES
JUMP TEST	SJ (cm)	39 ± 5	32 ± 3	**0.006**	1.73
	CMJ (cm)	42 ± 5	37 ± 6	0.105	0.90
	RSI (cm·ms^−1^)	0.92 ± 0.22	0.73 ± 0.11	0.052	1.12
WINGATE TEST	MPO (W)	671 ± 39	639 ± 70	0.306	0.55
	PPO (W)	823 ± 58	788 ± 86	0.367	0.47
	MPO (W∙kg^−1^)	8.6 ± 0.6	7.6 ± 0.8	**0.019**	1.40
	PPO (W∙kg^−1^)	10.6 ± 0.7	9.4 ± 1.1	**0.030**	1.28
	FS (%)	37 ± 11	35 ± 8	0.612	0.21
	SmO_2_ (%)	67 ± 7	69 ± 6	0.601	0.31
	(La^−^)_peak_ (mmol∙L^−1^_)_	15.3 ± 2.8	13.4 ± 4.3	0.325	0.52
INCREMENTAL TEST	VO_2max_ (ml∙kg^−1^∙min^−1^)	55.1± 5.	49.1± 4.	**0.022**	1.32
	Vpeak (km∙h^−1^)	17.0 ± 0.9	15.4 ± 1.2	**0.011**	1.49
	vVT1 (km∙h^−1^)	10.1 ± 0.6	9.9 ± 0.9	0.619	0.26
	vVT2 (km∙h^−1^)	15.1 ± 1.1	14.2 ± 1.1	0.128	0.82
	SmO_2_ (%)	64 ± 8	66 ± 10	0.733	0.22
STRENGTH TEST	1RM BP (kg)	105 ± 11	95 ± 21	0.279	0.58
	1RM BP (%BW)	1.4 ± 0.1	1.1 ± 0.2	**0.035**	1.20
	Pmax BP (W)	554 ± 104	505 ± 140	0.463	0.39
	Pmaxrel BP (W∙kg^−1^)	7.1 ± 1.4	6..0 ± 1.3	0.121	0.86
	1RM DFS (kg)	133 ± 19	128 ± 28	0.704	0.21
	1RM DFS (%BW)	1.7 ± 0.3	1.5 ± 0.2	0.164	0.75
	Pmax DFS (W)	1436 ± 300	1553 ± 529	0.616	0.27
	Pmaxrel DFS (W∙kg^−1^)	18.4 ± 3.7	18.1 ± 4.4	0.916	0.07

Data are Mean ± SD. Abbreviations: 1RM BP, one-repetition maximum bench press; 1RM DFS, one-repetition maximum deep full squat; CMJ, countermovement jump; ES, effect size; FS, fatigue slope; (La^−^)peak, lactate peak; MPO, mean power output; Pmax BP, maximum power in bench press; Pmax DFS, maximum power in deep full squat; PPO, peak power output; RSI, reactive strength index; SJ, squat jump; SmO_2_, muscle oxygen saturation; VO_2max_, maximum oxygen consumption; Vpeak, peak speed; vVT1, speed at ventilatory threshold 1; vVT2, speed at ventilatory threshold 2. Significant *p*-values are in bold.

**Table 3:** Relationship between the different physiological variables and the repetitions performed on each workout of the day.

Test	Variable	WOD 1	WOD 2	WOD 3	WOD 4	WOD 5
		r	r	r	r	r
JUMP TEST	SJ (cm)	0.29	**0.79** **	**0.77** **	**0.58** *	**0.82** **
	CMJ (cm)	0.05	**0.59** *	**0.56** *	0.43	**0.73** **
	RSI (cm·ms^−1^)	**0.52** *	**0.82** **	**0.60** *	**0.59** *	**0.63** *
WINGATE TEST	MPO (W)	**0.56** *	0.45	0.28	0.23	0.33
	PPO (W)	0.45	0.32	0.13	0.12	0.06
	MPO (W∙kg^−1^)	0.47	**0.77** **	**0.59** *	**0.66** **	**0.65** **
	PPO (W∙kg^−1^)	0.40	**0.64** *	0.45	**0.55** *	0.40
	FS (%)	−0.10	−0.18	−0.10	−0.04	−0.35
	SmO_2_ (%)	0.14	−0.32	−0.05	−0.23	−0.09
	(La^−^)_peak_ (mmol∙L^−1^)	0.14	**0.58** *	0.22	0.31	0.43
INCREMENTAL TEST	VO_2max_ (ml∙kg^−1^∙min^−1^)	**0.63** *	0.46	0.47	**0.54** *	0.31
	Vpeak (km∙h^−1^)	**0.53** *	**0.64** *	**0.62** *	**0.56** *	0.43
	vVT1(km∙h^−1^)	−0.08	0.20	0.07	0.05	−0.01
	vVT2 (km∙h^−1^)	0.21	0.47	0.37	0.27	0.07
	SmO_2_ (%)	0.14	−0.32	−0.05	−0.23	−0.09
STRENGTH TEST	1RM BP (kg)	0.36	0.26	0.41	0.17	0.29
	1RM BP (%BW)	0.36	**0.53** *	**0.66** **	0.46	**0.53** *
	Pmax BP (W)	**0.60** *	0.38	0.32	0.14	0.20
	Pmax BP (W∙kg^−1^)	**0.63** *	**0.63** *	**0.54** *	0.38	0.40
	1RM DFS (kg)	0.40	0.31	0.32	0.18	0.46
	1RM DFS (%BW)	0.42	**0.64** **	**0.61** *	**0.52** *	**0.77** **
	Pmax DFS (W)	0.45	0.10	−0.01	−0.09	−0.07
	Pmax DFS (W∙kg^−1^)	**0.56** *	0.38	0.19	0.13	0.09

Abbreviations: 1RM BP, one-repetition maximum bench press; 1RM DFS, one-repetition maximum deep full squat; CMJ, countermovement jump; FS, fatigue slope; (La^−^)peak, lactate peak; MPO, mean power output; Pmax BP, maximum power bench press; Pmax DFS, maximum power deep full squat; PPO, peak power output; RSI, reactive strength index; SJ, squat jump; SmO_2_, muscle oxygen saturation; VO_2max_, maximum oxygen consumption; Vpeak, peak speed; vVT1, speed at ventilatory threshold 1; vVT2, speed at ventilatory threshold 2. Score performance was considered as the number of repetitions attained in each workout of the day (WOD). A higher number of repetitions indicates a better performance. Significant correlations are in bold (* *p* < 0.05, ** *p* < 0.01).

**Table 4:** Relationship between the different physiological variables and the position (i.e., ranking within the group) for each workout of the day and overall CrossFit performance.

Test	Variable	WOD 1	WOD 2	WOD 3	WOD 4	WOD 5	Overall Performance
		ρ	ρ	ρ	ρ	ρ	r
JUMP TEST	SJ (cm)	−0.32	**−0.69** **	**−0.73** **	**−0.69** **	**−0.73** **	**−0.70** **
	CMJ (cm)	**−0.56** *	**−0.55** *	**−0.51** *	**−0.55** *	**−0.65** **	**−0.63** *
	RSI ( cm·ms^−1^)	**−0.59** *	**−0.89** **	**−0.70** **	**−0.72** **	**−0.62** *	**−0.79** **
WINGATE TEST	MPO (W)	**−0.58** *	−0.41	−0.25	−0.32	−0.32	−0.39
	PPO (W)	−0.49	−0.35	−0.16	−0.26	−0.09	−0.27
	MPO (W∙kg^−1^)	−0.48	**−0.74** **	**−0.63** **	**−0.69** **	**−0.74** **	**−0.68** **
	PPO (W∙kg^−1^)	**−0.63** *	**−0.62** *	−0.52	**−0.64** *	**−0.59** *	**−0.63** *
	FS (%)	−0.06	0.70	0.01	−0.07	0.13	0.02
	SmO_2_ (%)	−0.24	0.30	−0.06	−0.20	−0.00	0.05
	(La^−^)_peak_ (mmol∙L^−1^)	−0.12	**−0.53** *	−0.34	−0.44	−0.38	−0.35
INCREMENTAL TEST	VO_2max_ (ml∙kg^−1^∙min^−1^)	**−0.60** *	−0.47	−0.51	**−0.55** *	**−0.63** *	**−0.62** *
	Vpeak (km∙h^−1^)	−0.34	−0.45	**−0.55** *	**−0.57** *	**−0.71** **	**−0.67** **
	vVT1(km∙h^−1^)	−0.00	0.09	0.10	0.03	−0.23	−0.00
	vVT2 (km∙h^−1^)	−0.25	−0.38	−0.39	−0.34	−0.28	−0.39
	SmO_2_ (%)	−0.21	0.21	−0.04	0.13	−0.02	−0.02
STRENGTH TEST	1RM BP (kg)	−0.20	−0.15	−0.27	−0.19	−0.13	−0.26
	1RM BP (%BW)	−0.28	−0.50	**−0.71** **	**−0.65** *	**−0.65** **	**−0.64** **
	Pmax BP (W)	−0.45	−0.22	−0.15	−0.15	−0.22	−0.29
	Pmax BP (W∙kg^−1^)	−0.50	−0.44	−0.42	−0.41	**−0.53** *	**−0.52** *
	1RM DFS (kg)	−0.38	−0.39	−0.38	−0.41	**−0.57** *	−0.50
	1RM DFS (%BW)	−0.41	**−0.56** *	**−0.54** *	**−0.60** *	**−0.78** **	**−0.66** **
	Pmax DFS (W)	−0.35	−0.25	−0.02	−0.01	0.00	−0.12
	Pmax DFS (W∙kg^−1^)	−0.34	−0.34	−0.12	−0.12	−0.17	−0.22

Abbreviations: 1RM BP, one-repetition maximum bench press; 1RM DFS, one-repetition maximum deep full squat; CMJ, countermovement jump; FS, fatigue slope; (La^−^)peak, lactate peak; MPO, mean power output; Pmax BP, maximum power bench press; Pmax DFS, maximum power deep full squat; PPO, peak power output; RSI, reactive strength index; SJ, squat jump; SmO_2_, muscle oxygen saturation; VO_2max_, maximum oxygen consumption; Vpeak, peak speed; vVT1, speed at ventilatory threshold 1; vVT2, speed at ventilatory threshold 2. Performance was considered as the position (1–15) attained within the group in each workout of the day (WOD), and overall performance is the sum of the points achieved in each WOD for each athlete. A higher position indicates a worse performance. Significant correlations are in bold (* *p* < 0.05, ** *p* < 0.01).

## References

[1] Claudino J.G., Gabbett T.J., Bourgeois F., Souza H.D.S., Miranda R.C., Mezêncio B., Soncin R., Filho C.A.C., Bottaro M., Hernandez A. (2018). CrossFit Overview: Systematic Review and Meta-analysis. Sports Med. Open.

[2] Butcher S., Neyedly T.J., Horvey K.J., Benko C.R. (2015). Do physiological measures predict selected CrossFit® benchmark performance?. Open Access. J. Sports Med..

[3] Martínez-Gómez R., Valenzuela P.L., Barranco-Gil D., Moral-González S., García-González A., Lucia A. (2019). Full-Squat as a Determinant of Performance in Cross Fit. Int. J. Sports Med..

[4] Bellar D., Hatchett A., Judge L.W., E Breaux M., Marcus L. (2015). The relationship of aerobic capacity, anaerobic peak power and experience to performance in CrossFit exercise. Boil. Sport.

[5] Dexheimer J.D., Schroeder E.T., Sawyer B.J., Pettitt R.W., Aguinaldo A.L., Torrence W.A. (2019). Physiological Performance Measures as Indicators of CrossFit® Performance. Sports.

[6] Driss T., Vandewalle H. (2013). The Measurement of Maximal (Anaerobic) Power Output on a Cycle Ergometer: A Critical Review. Biomed Res. Int..

[7] Amann M., Subudhi A.W., Foster C. (2006). Predictive validity of ventilatory and lactate thresholds for cycling time trial performance. Scand. J. Med. Sci. Sports.

[8] Joyner M.J., Coyle E.F. (2007). Endurance exercise performance: The physiology of champions. J. Physiol..

[9] LoTurco I., Pereira L., Abad C., Tabares F., Moraes J.E., Kobal R., Kitamura K., Nakamura F.Y. (2016). Bar velocities capable of optimising the muscle power in strength-power exercises. J. Sports Sci..

[10] LoTurco I., Pereira L., Abad C.C.C., D’Angelo R.A., Fernandes V., Kitamura K., Kobal R., Nakamura F.Y. (2015). Vertical and Horizontal Jump Tests Are Strongly Associated With Competitive Performance in 100-m Dash Events. J. Strength Cond. Res..

[11] LoTurco I., Pereira L., Kobal R., Maldonado T., Piazzi A.F., Bottino A., Kitamura K., Abad C.C.C., De Arruda M., Nakamura F.Y. (2016). Improving Sprint Performance in Soccer: Effectiveness of Jump Squat and Olympic Push Press Exercises. PLoS ONE.

[12] Feito Y., Giardina M.J., Butcher S., Mangine G.T. (2019). Repeated anaerobic tests predict performance among a group of advanced CrossFit-trained athletes. Appl. Physiol. Nutr. Metab..

[13] Pérez-Castilla A., Piepoli A., Delgado-García G., Garrido-Blanca G., García-Ramos A. (2019). Reliability and Concurrent Validity of Seven Commercially Available Devices for the Assessment of Movement Velocity at Different Intensities During the Bench Press. J. Strength Cond. Res..

[14] Conceição F., Fernandes J., Lewis M.G.C., González-Badillo J.J., Jiménez-Reyes P. (2015). Movement velocity as a measure of exercise intensity in three lower limb exercises. J. Sports Sci..

[15] González-Badillo J.J., Sánchez-Medina L. (2010). Movement Velocity as a Measure of Loading Intensity in Resistance Training. Int. J. Sports Med..

[16] Garcia-Ramos A., Jaric S. (2018). Feasibility of the Two- Point Method for Determining the One- Repetition Maximun in the Bench Press Exercise. Strength Cond. J..

[17] García-Ramos A., Pestaña-Melero F.L., Pérez-Castilla A., Rojas F.J., Haff G.G. (2018). Differences in the Load–Velocity Profile Between 4 Bench-Press Variants. Int. J. Sports Physiol. Perform..

[18] Glatthorn J.F., Gouge S., Nussbaumer S., Stauffacher S., Impellizzeri F.M., A Maffiuletti N. (2011). Validity and Reliability of Optojump Photoelectric Cells for Estimating Vertical Jump Height. J. Strength Cond. Res..

[19] Jones A.M., Doust J.H. (1996). A 1% treadmill grade most accurately reflects the energetic cost of outdoor running. J. Sports Sci..

[20] Lucia A., Pardo J., Durántez A., Hoyos J., Chicharro J.L. (1998). Physiological differences between professional and elite road cyclists. Int. J. Sports Med..

[21] Farzam P., Starkweather Z., Franceschini M.A. (2018). Validation of a novel wearable, wireless technology to estimate oxygen levels and lactate threshold power in the exercising muscle. Physiol. Rep..

[22] Bonaventura J.M., Sharpe K., Knight E., Fuller K.L., Tanner R.K., Gore C.J. (2015). Reliability and Accuracy of Six Hand-Held Blood Lactate Analysers. J. Sports Sci. Med..

[23] Tanner R.K., Fuller K.L., Ross M.L.R. (2010). Evaluation of three portable blood lactate analysers: Lactate Pro, Lactate Scout and Lactate Plus. Eur. J. Appl. Physiol..

[24] CrossFit Games: Open 2019. https://games.crossfit.com/workouts/open/2019.

[25] Borszcz F.K., Tramontin A.F., Costa V.P. (2019). Is the Functional Threshold Power Interchangeable With the Maximal Lactate Steady State in Trained Cyclists?. Int. J. Sports Physiol. Perform..

[26] LoTurco I., Pereira L., Moraes J.E., Kitamura K., Abad C.C.C., Kobal R., Nakamura F.Y. (2017). Jump-Squat and Half-Squat Exercises: Selective Influences on Speed-Power Performance of Elite Rugby Sevens Players. PLoS ONE.

[27] Bar-Or O. (1987). The Wingate anaerobic test. An update on methodology, reliability and validity. Sports Med..

[28] Hopkins W. (2007). A spreadhseet to compare means of two groups. Sports Sci..

[29] Hopkins W.G., Marshall S., Batterham A., Hanin J. (2009). Progressive Statistics for Studies in Sports Medicine and Exercise Science. Med. Sci. Sports Exerc..

[30] Comfort P., Stewart A., Bloom L., Clarkson B. (2014). Relationships Between Strength, Sprint, and Jump Performance in Well-Trained Youth Soccer Players. J. Strength Cond. Res..

[31] Hoffman J.R., Epstein S., Einbinder M., Weinstein Y. (2000). A Comparison Between the Wingate Anaerobic Power Test to Both Vertical Jump and Line Drill Tests in Basketball Players. J. Strength Cond. Res..

[32] Andersen E., Lockie R., Dawes J.J. (2018). Relationship of Absolute and Relative Lower-Body Strength to Predictors of Athletic Performance in Collegiate Women Soccer Players. Sports.

